# Depressive and anxiety symptoms, and their associated factors among adult, visually impaired follow-up patients attending the Hawassa University Comprehensive Specialized Hospital Eye Care and Training Center, South Ethiopia, 2024

**DOI:** 10.1371/journal.pone.0326117

**Published:** 2025-07-02

**Authors:** Beza Feleke Wube, Yezinash Addis, Biruk Fanta Alemayehu, Seblework Adugna Etsub, Biruk Lelisa Eticha

**Affiliations:** 1 Department of Ophthalmology and Optometry, College of Medicine and Health Sciences, Comprehensive Specialized Hospital, Hawassa University, Hawassa, Ethiopia; 2 Department of Optometry, College of Medicine and Health Sciences, Comprehensive Specialized Hospital, University of Gondar, Gondar, Ethiopia; 3 Department of Psychiatry, College of Medicine and Health Sciences, Comprehensive Specialized Hospital, University of Gondar, Gondar, Ethiopia; 4 Department of Ophthalmology, College of Medicine and Health Sciences, Comprehensive Specialized Hospital, University of Gondar, Gondar, Ethiopia; Wuhan Mental Health Centre, CHINA

## Abstract

**Background:**

Although the burden of depressive and anxiety symptoms among visually impaired adults has become a serious current health issue, relatively little is known in the study setting and in Ethiopia as well. This study aimed to assess the magnitude of depressive symptoms, anxiety, and their associated factors among adult, visually impaired follow-up patients of the Hawassa University Comprehensive Specialized Hospital Eye Care and Training Center, South Ethiopia, 2024.

**Methods:**

From 10 June to 2 August 2024, an institution-based cross-sectional study was conducted on 518 study subjects using a stratified sampling technique. Data were collected using a pretested, structured, and interviewer-administered questionnaire. The Hospital Anxiety Depressive symptoms Scale was used for assessment of depressive and anxiety symptoms among the study subjects in this study. This validated tool is composed of 14 items (7 for depressive symptoms and 7 for anxiety). The clinical and visual impairment related characteristics was extracted from their medical recording chart in daily basis. The statistical package for social science version 26 was used to analyze the data. Binary logistic regression was fitted. Variables with a p-value less than 0.05 were considered statistically significant.

**Results:**

From 518 visually impaired follow-up patients who participated in the study, about 34.9% (95% CI: 29.3–41.3) and 39.4% (95% CI: 35.1–43.8) experienced some level of depressive symptoms and anxiety symptoms, respectively. Social support, levels of visual impairment, a history of eye surgery, the pattern of vision loss, and patient satisfaction with an eye care service were variables significantly associated with both depressive and anxiety symptoms.

**Conclusion and recommendations:**

Relatively higher burdens of depressive and anxiety symptoms were observed in study subjects. Screening common mental disorders during assessment of visual impairment is helpful. To enhance people's general health, public health programs must primarily target the most vulnerable populations.

## Introduction

Visual Impairment (VI) refers to a functional limitation of the eye or visual system due to a disorder or disease that results in poor vision in either or both eyes [1]. According to the International Classification of Disease (ICD) 11^th^ definition, it is defined as presenting distance visual acuity (PVA) worse than 6/12 in the better eye [2]. It causes significant impairments to an individual's ability to function independently, which leads to disabilities. There is a visual handicap, and these limitations limit an individual's social and personal independence [3].

According to the World Health Organization (WHO) report, 2.2 billion people are living with some form of VI globally [4]. Global rates of blindness are predicted to triple in the next 30 years, whereas incidences of moderate VI are predicted to double [5]. It affects about 285 million individuals, of whom 266.4 million were adults aged 18 years and above [4]. Over 80% of VI is found among the underserved population in low- and middle-income countries [4]. In Sub-Saharan Africa, the prevalence of VI was 5.3% [6]. In Ethiopia, it ranges from 10.3% in Addis Ababa, central Ethiopia [7], to 37.58% in Northern Ethiopia [8].

Depressive symptoms are characterized by sadness, loss of interest or pleasure, feelings of guilt or low self-worth, disturbed sleep or appetite, feelings of tiredness, and poor concentration. It can be long-lasting or recurrent, substantially impairing an individual’s ability to function at work or school or cope with daily life [9]. Anxiety refers to a group of mental disorders characterized by feelings of fear [9]. Depression and anxiety in visually impaired adults are the most common psychiatric disorders and carry a high burden in terms of treatment costs, the effect on the ability to cope with daily activities, and on families and careers, as well as a loss of workplace productivity [10,11].

As compared to healthy population visually impaired individuals were two to five fold more vulnerable to symptomatic depression [12]. Globally, the results of meta-analysis indicated that 1 in 4 patients with VI who attended eye care services experienced at least significant depressive symptoms or more advanced disorder [13]. Additionally studies conducted on different age group adults suffering from different ocular conditions and related visual conditions experienced from 10 to 61% burden of depressive symptoms [12,14]. On the other hand, a meta-analysis and systematic review involving 91 studies conducted on study subjects suffering from a range of chronic ocular conditions causing VI revealed 21.2% to 53.5% prevalence range of anxiety symptoms [15]. Besides, the prevalence of anxiety among visually impaired individuals ranges from 6.5% to 77.5% [16–18]. In Africa, the range of prevalence of depressive and anxiety symptoms among visually impaired patients interviewed in different health centers was 8.7% [19] to 50% [20] and 10.4% [21] to 53.2% [20], respectively. Besides, studies conducted in Ethiopia reported quite variety of burden of depressive symptoms, ranging from 26.7% [22] to 30.6% [23].

Age [24,25], marital status [12,26], sex [27], illiteracy [12], residence [27], religion [12], living status [23,28], social support [26], additional physical impairments [25], comorbidity [27], VI duration [26], VI level [12,26], pattern of vision loss [25], VI cause [23], substance use [26], and emotional and personality [12] were significantly associated with depressive symptoms. On the other hand, a significant association was observed between anxiety symptoms and age [29,30], marital status [30], duration of VI [31], unilateral VI [32], and severity of VI [29,30].

However, there are efforts being made to decrease the impact of common mental disorders on individuals’ well-being; limited data were unveiled regarding the current magnitude of depressive and anxiety symptoms among visually impaired patients yet. Hence, knowing the prevalence of depressive and anxiety symptoms among visually impaired patients helps to assess the impacts of this national eye care strategic plan and recast it based on the findings implicating at least minimum manifestations with their respective levels of symptoms. Moreover, it could be considered as evidence for eye care professionals and policymakers to obtain focused, mobilized, and essential resources for the implementation of suitable administrative and therapeutic procedures, early detection, and referral for depressive and anxiety symptoms.

Such study focusing on the early or progressive symptoms of depression and anxiety helps to anticipate efficient preventive and rehabilitative management that could equip the health care system act at the grass root level of the problem. Therefore, the aim of this study was to assess the prevalence and associated factors of depressive and anxiety symptoms among adult, visually impaired follow-up patients of the Hawassa University Comprehensive Specialized Hospital Eye Care and Training Center (HUCSH-ECTC), South Ethiopia.

## Materials and methods

### Study design, area, and period

An institution-based cross-sectional study was conducted at HUCSH-ECTC in Hawassa City, South Ethiopia, from June 10 to August 2, 2024. In the area, it is the largest and sole tertiary facility serving around 16 million people in the Sidama Regional State and catchment areas that include Southern Ethiopia People Regional State and the neighboring Oromia Region [33]. The eye care and training center is comparted from both outpatient and inpatient departments and different units that provide medical, surgical, laser, and refractive therapy [34]. The center provides comprehensive eye care services for all new and follow-up visually impaired patients.

### Source and study population

All adult, visually impaired patients who were under follow-up at HUCSH-ECTC for at least the last month.

### Eligibility criteria

All adult, visually impaired patients who were under follow-up at HUCSH-ECTC for at least the last month were included in the study. However, adult visually impaired patients who could not give a response, including those with medical emergencies, hearing, and speaking problems, were excluded from the study.

### Sample size determination

#### For primary objectives.

The sample size for prevalence was determined using a single population proportion formula, taking into account 0.267 and 0.295 proportions of depressive and anxiety symptoms taken from researches conducted in Ethiopia [22] and Nigeria [29], respectively. Furthermore, the sample size calculations stated below considered a 5% significance level and a 4% margin of error in common.


$$n_{\left(Depressive\;symptoms\right)}\;=\;\frac{\left(Z_{\alpha /2})\;2*\;P\;(1-P\right)}{d^{2}}=\;\frac{\left(1.96)\;2*\;0.267\;(1-0.267\right)}{0.04^{2}}$$

$$n_{\left(Anxiety\;symptoms\right)}\;=\;\frac{\left(Z_{\alpha /2})\;2*\;P\;(1-P\right)}{d^{2}}=\;\frac{\left(1.96)\;2\text{* }0.295\;(1-0.295\right)}{0.04^{2}}$$

The final sample size calculated for the primary objective considering depressive and anxiety symptoms became 469.9 and 499.3, respectively.

Where;

n = Sample size

Z = Z statistics for 95% level of confidence = 1.96

P = Proportion of depressive and anxiety symptoms = 0.267 [22] and 29.5 [29], respectively

d = Margin of error = ±4%

#### For secondary objectives.

Level of VI, duration of illness, and social support were consistent factors of depressive and anxiety symptoms among visually impaired adults in the study done in Ethiopia [17] and Nigeria [16], respectively. The Epidemiological Information version 7.2.6.0 computer software at a 95% confidence level and 80% power was used to determine the maximum sample size for the secondary objective, 210 (Table 1).

**Table 1:** Sample size determination for associated factors of depressive and anxiety symptoms among adult visually impaired follow-up patients attending HUCSH-ECTC, South Ethiopia, 2024.

No	Factors	Depressive/ anxiety symptoms		Total	COR	Sample size
		Yes	No			
	**Factors associated with depressive symptoms**					
1	Level of VI					
	Mild	19	104	123	1.00	132
	Severe	50	80	130	3.42	
2	Duration of illness					
	<1 year	6	141	147	1.00	54
	>1 years	116	187	303	14.58	
3	Social support					
	Poor	75	108	183	5.82	76
	Strong	8	67	75	1.00	
	**Factors associated with anxiety symptoms**					
1	Level of VI					
	Mild	28	56	84	1.00	216
	Severe	52	46	98	2.26	

No – Number; VI – Visual Impairment; COR – Crude Odds Ratio.

To meet both objectives of this study and after bearing in mind the 10% nonresponse rate, we took the maximum sample size, 550 (the sample size for the primary objective), as the final sample size.

### Sampling technique and procedure

The study participants were selected from three follow-up clinics found in HUCSH-ECTC. Out of the total of 1,122 expected average number of visually impaired patients attending these follow-up clinics, 550 study participants were selected using the stratified sampling technique (S1 Fig).

Taking the account of patient flow of each clinic, the total sample size was allocated proportionally. Once the allocation was done, the sampling interval for each clinic was calculated, and the value was determined to be two. We used the lottery method to draw the first sample of the first two participants and continued with every other participant. When the number of patients available was equal to or less than the average number of samples for that specific day and clinic, we used all of the eligible patients in that specific clinic as study participant.

### Study variables

**Dependent variables:** Depressive symptoms and anxiety symptoms

**Independent variables:**

**Socio-demographic and economic factors**: Age, sex, residence, religion, marital status, educational level, occupation, average family monthly income, family size, valid health insurance, living circumstance, and social support

**VI-related factors**: Level of VI, pattern of vision loss, current status of VI, and main cause of VI

**Clinical factors**: Duration of VI, history of eye surgery, history of medical illness, family history of mental illness, substance use, additional physical and sensory impairment, and patient satisfaction

### Operational definitions

**Depressive symptoms:** An individual scored the total score of ≥ 8 points out of a possible 21 subscale points of the depressive symptoms component of the Hospital Anxiety Depressive symptoms Scale (HADS) was deemed as experiencing depressive symptoms [35].

**Anxiety symptoms:** A study participant that got a total subscale score of ≥8 points out of a possible 21 points of the HADS anxiety subscale was considered as experiencing anxious symptoms [35]. Regarding the levels of anxiety symptoms, study subjects’ anxiety symptoms was leveled as mild, moderate, and severe, based on the individuals’ total score from 8 to 10, 11–15, and 16–21, respectively.

**Visual impairment:** Defined as per the ICD 11^th^ disease classification [36].

- **Mild VI**: PVA worse than 6/12–6/18 in the better eye
- **Moderate VI**: PVA worse than 6/18–6/60 in the better eye
- **Severe VI**: PVA worse than 6/60 in the better eye

**Social support**: Participants in the study were classified as acquiring weak, moderate, and strong social support if their scores on the Oslo Social Support Scale (OSSS) ranged from 3 to 8, 9–11, and 12–14 [37].

**Satisfied:** respondents who mark an overall mean score of 60.00% to 100% or fulfill the overall mean score (54) or more on the given items to measure satisfaction [38].

**Adult:** A person whose age was ≥ 18 years old [39].

**First relative family:** Mother, father, sister, brother, husband, and wife of the study participant.

**Current substance user:** An individual who has a history of specified substance use for non-medical purposes in the last 12 months was identified as a current substance user [40].

### Data collection tool and procedures

Under the eyes of a supervisor, three senior optometrists collected the data using a pretested and structured interviewer-administered Amharic, Afan-Oromo, and Sidaamu-Afoo version questionnaire. The questionnaire contained the study subjects’ socio-demographic and economic characteristics, clinical and VI-related characteristics, and the so-called HADS-14 questionnaire used for measuring depressive and anxiety symptoms [35]. The questionnaire prepared in Kobo Collect version 2021.2.4 was used for the face-to-face interview and chart review. The HADS-14 is a validated and reliable instrument designed to detect the states of anxiety and depressive symptoms in a hospital outpatient setting. The HADS questionnaire has seven items each for depressive and anxiety symptoms subscales. Each item was measured with a 4-point Likert scale ranging from 0 to 3. The scores of each subscale were summed separately, with higher scores denoting the highest anxiety or depressive symptoms. A total subscale score of ≥ 8 points out of a possible 21 denotes considerable anxiety or depressive symptoms [35]. The item’s reliability was checked by calculating Cronbach’s alpha value (α = 0.993) using a pre-test at Adare General Hospital. The higher validity, reliability and internal consistency of the items of the HADS questionnaire influences us to use to assess the presence of depressive and anxiety symptoms among the study subjects. Social support was assessed by using the OSSS, which had three items used to assess the level of the participant’s social support. The scale ranged from 3 to 14, and the scores 3–8, 9–11, and 12–14 showed “poor,” “moderate,” and “strong” social support [37], respectively. Moreover, patient satisfaction towards an eye care service obtained was assessed by the Patient Satisfaction Questionnaire (PSQ-18) [38].

### Data quality assurance

The quality of the data was ensured by using a pretested structured questionnaire in Amharic, Afan-Oromo, and Sidaamu-Afoo. To ensure reliability and consistency, questions were translated by language experts into Amharic, Afan-Oromo, and Sidaamu-Afoo before being retranslated back into English. Once a pretest was done on 5% (27) of the sample size at Adare General Hospital, appropriate modification was done based on the feedback acquired. Besides, training was adequately given for the data collectors using standardized questionnaire before data collection. Furthermore, to ensure the quality of the data, daily supervision went on during the data collection period. Prior to analysis, the principal investigator cleaned up, crosschecked the gathered data, and verified that it was accurate, complete, and clear.

### Data processing and analysis

The data was entered into Kobo Collect version 2021.2.4 and was exported and checked, cleaned, and analyzed by using Stata version 17. Descriptive statistics such as proportion and frequency were calculated. A bivariable binary logistic regression was used to assess the effects of all independent variables on the prevalence of depressive and anxiety symptoms and to select a candidate variable for the multivariable analysis. To find the final associated factors of the outcome variables, a multivariable binary logistic regression was used for variables with a p-value < 0.20 in bivariable binary logistic regression. Using the Hosmer-Lemeshow model fitness test, the model fitness for depressive and anxiety symptoms was validated with p-values of 0.85 and 0.77, respectively. Likewise, the absence of multicollinearity between predictor variables was checked using the variance inflation factor. The Adjusted Odds Ratio (AOR) with a 95% Confidence Interval (CI) was used to determine the strength of association of actual associated factors of depressive and anxiety symptoms, and the P-value < 0.05 was considered statistically significant.

### Ethical considerations

First, ethical clearance with the reference number of SOM/1456/2024 was obtained from the University of Gondar College of Medicine and Health Sciences, School of Medicine Ethical Review Committee. Then, an official support letter and approval were obtained from the Department of Optometry and HUCSH-ECTC, respectively. Following a detailed explanation regarding the purpose, benefit, and risk of the study, verbal informed consent was acquired from each of the study participants. Participants were given full freedom to refuse to participate and withdraw from the study at any point of the study process. To ensure confidentiality, the questionnaires were coded, and the names of respondents were not included in the questionnaire. Data collected in this study were used strictly for research purposes and secured by coding the data and maintaining it during the data collection and analysis. Furthermore, those screened positive for depressive and anxiety symptoms were linked to the psychiatric outpatient department for further evaluation. Finally, it is our pleasure to reassure that the study was conducted with strict adherence to the World Medical Association’s Declaration of Helsinki.

## Results

### Socio-demographic and economic characteristics of the study participants

This research involved 518 adult, visually impaired, follow-up study participants, with a 94.18% response rate. The median age of the participants was 55 ± 27 Interquartile Range (IQR). Approximately sixty percent of the study subjects, 310 (59.8%) and 307 (59.3%), were male and married, respectively (Table 2).

**Table 2:** Socio-demographic and economic characteristics of adult visually impaired follow-up patients attending HUCSH-ECTC, South Ethiopia, 2024 (n = 518).

Variables	Frequency	Percentages (%)
Age in year		
18-35	124	23.94
36-50	120	23.17
51-65	141	27.22
> 65	133	25.67
Sex		
Male	310	59.85
Female	208	40.15
Religion		
Orthodox	239	46.14
Muslim	51	9.85
Protestant	211	40.73
Others*	17	3.28
Marital status		
Currently married	307	59.26
Currently not married	211	40.74
Residence		
Urban	321	61.97
Rural	197	38.03
Educational level		
Non-formal education	171	33.00
Formal education	347	67.00
Occupation		
Farmer	129	24.90
Government employee	99	19.11
Self-employer	110	21.24
House wife	64	12.36
Merchant	71	13.71
Retired	21	4.05
Others**	24	4.63
Average family monthly income (in ETB)		
< 4000	99	19.11
4000-6000	222	42.86
6000-8000	82	15.83
> 8000	115	22.20
Family size		
3-4	163	31.47
5-6	293	56.56
> 6	62	11.97
Valid health insurance		
Yes	274	52.90
No	244	47.10
Living circumstance		
First relative family	414	79.92
Alone	51	9.85
Relative	53	10.23
Social support		
Poor	153	29.54
Moderate	134	25.87
Strong	231	44.59

ETB – Ethiopian Birr; Others* (catholic and Adventist); Others** (students and non-employed)

### Clinical and VI-related characteristics of the study participants

Of the respondents, 247 (47.7%) had a moderate level of VI and above eighty percent, 429 (82.8%), had a stable nature of VI. Besides, about two-thirds of the study subjects, 330 (63.7%), had a progressive pattern of vision loss. Regarding the main cause of VI, with the frequency of 182 (35.1%), 142 (27.4%), and 93 (18.0%), cataract, refractive error, and glaucoma were the top three main causes of VI (Table 3).

**Table 3:** Clinical and visual impairment related characteristics of adult visually impaired follow-up patients attending HUCSH-ECTC, South Ethiopia, 2024 (n = 518).

Variables	Frequency	Percentages (%)
Level of VI		
Mild	153	29.54
Moderate	247	47.68
Severe	118	22.78
Duration of illness		
< 1 year	113	21.81
1–2 years	88	16.99
2–5 years	216	41.70
> 5 years	101	19.50
Pattern of vision loss		
Sudden	188	36.29
Progressive	330	63.71
Current status of VI		
Stable	429	82.82
Progressive	89	17.18
Main cause of VI		
Cataract	182	35.14
Glaucoma	93	17.95
Refractive error	142	27.41
Corneal opacity	28	5.41
Keratitis	28	5.41
Uveitis	20	3.86
Others*	25	4.82
History of eye surgery		
Yes	100	19.31
No	418	80.69
History of medical illness		
Yes	139	26.83
No	379	73.17
Family history of mental illness		
Yes	11	2.12
No	507	97.88
Substance-use		
Yes	16	3.09
No	502	96.91
Additional physical and sensory impairment		
Yes	17	3.28
No	501	96.72
Patient satisfaction		
Satisfied	314	60.62
Unsatisfied	204	39.38

VI – Visual Impairment; Other* (Diabetic retinopathy, Retinal detachment, Age-related macular degeneration, Retinal vein occlusion, Vitreous hemorrhage, Retinitis pigmentosa, and Optic atrophy)

### Prevalence of depressive and anxiety symptoms

In this study, the prevalence of depressive and anxiety symptoms among adult, visually impaired follow-up patients was 181 (34.9%) (95% CI: 29.3, 41.3) and 204 (39.4%) (95% CI: 35.1, 43.8), respectively.

From individuals who had depressive symptoms, study subjects experienced a moderate level of the condition comparted almost the two-thirds (64.09%) of total participants. By the same token, about three-fourths (75%) of the respondents suffered from the moderate level of anxiety symptoms. Moreover, only 8.82% of the respondents were confirmed to be severely anxious (S2 Fig).

### Factors associated with depressive symptoms

The bivariate binary logistic regression at a p-value < 0.2 unveiled that age, residence, marital status, educational status, average family monthly income, living circumstance, family size, level of VI, duration of VI, current status of VI, pattern of vision loss, additional impairment, history of eye surgery, history of medical illness, social support, and patient satisfaction were eligible to undergo multivariable binary logistic regression of depressive symptoms.

The results of multivariate logistic regression analysis indicated that age, marital status, vision loss pattern, history of eye surgery, level of VI, social support, and patient satisfaction with service provided were significantly associated with depressive symptoms at a p-value < 0.05 (Table 4).

**Table 4:** Factors associated with depressive symptoms among adult visually impaired follow-up patients attending HUCSH-ECTC, South Ethiopia, 2024 (n = 518).

Variable	Depressive symptoms		COR (95%)	AOR (95%)	P-value
	No	Yes			
Age					
** **18-35	88	36	1.00	1.00	
** **36-50	94	26	0.68(0.38-1.21)	2.60(1.13-6.02) *	0.046
** **51-65	82	59	1.76(1.05-2.94)	4.09(1.79-9.36) **	0.002
** ** > 65	73	60	2.10(1.20-3.37)	1.99(0.87-4.59)	0.087
Residence					
** **Urban	231	90	1.00	1.00	
** **Rural	106	91	2.20(1.52-3.19)	0.87(0.39-1.94)	0.156
Marital status					
** **Currently married	239	68	1.00	1.00	
** **Currently not married	98	113	4.05(2.77-5.94)	5.95(3.37-10.50) ***	0.000
Living circumstance					
** **First relative family	283	131	1.00	1.00	
** **Alone	35	16	.99(0.53-1.85)	0.47(0.18-1.19)	0.245
** **Relative	19	34	3.87(2.13-7.03)	0.45(0.17-1.23)	0.371
Educational status					
Non-formal education	91	80	2.14(1.47-3.13)	0.79(0.37-1.70)	0.183
Formal education	246	101	1.00	1.00	
Average family monthly income (in ETB)					
** ** < 4000	47	52	4.20(2.31-7.63)	2.33(0.88-6.15)	0.401
** **4000-6000	138	84	2.31(1.37-3.90)	1.48(0.72-3.06)	0.234
** **6000-8000	61	21	1.31(0.67-2.55)	2.24(0.94-5.30)	0.523
** ** > 8000	91	24	1.00	1.00	
Family size					
** **3-4	110	53	1.00	1.00	
** **5-6	193	100	1.08(0.72-1.62)	0.84(0.47-1.47)	0.292
** ** > 6	34	28	1.71(0.94-3.11)	0.93(0.41-2.12)	0.126
Duration of VI					
** ** < 1 year	78	35	1.00	1.00	
** **1–2 years	62	26	0.94(0.51-1.72)	1.07(0.48-2.41)	0.333
** **2–5 years	142	74	1.16(0.71-1.89)	1.38(0.70-2.73)	0.464
** ** > 5 years	55	46	1.86(1.07-3.26)	2.12(0.92-4.90)	0.763
Pattern of vision loss					
** **Sudden	96	92	2.60(1.78-3.78)	3.51(2.04-6.03) ***	0.000
** **Progressive	241	89	1.00	1.00	
Current status of VI					
** **Stable	272	157	1.00	1.00	
** **Progressive	65	24	0.64(0.39-1.06)	0.32(0.16-1.66)	0.068
Additional physical and sensory impairment					
** **Yes	5	12	4.72(1.63-13.60)	1.10(0.22-5.31)	0.616
** **No	332	169	1.00	1.00	
History of eye surgery					
** **Yes	45	55	2.83(1.81-4.42)	3.60(1.93-6.74) ***	0.000
** **No	292	126	1.00	1.00	
Any medical illness					
** **Yes	79	60	1.62(1.09-2.41)	1.59(0.84-3.01)	0.296
** ** No	258	121	1.00	1.00	
Level of VI					
** **Mild	116	37	1.00	1.00	
** **Moderate	178	69	1.22(0.77-1.93)	1.12(0.62-2.02)	
** **Severe	43	75	5.47(3.23-9.26)	2.98(1.43-6.23) **	0.003
Social support					
** **Poor	61	92	8.45(5.21-13.70)	6.73(3.65-12.42) ***	0.000
** **Moderate	80	54	3.78(2.30-6.22)	4.04(2.22-7.36) ***	0.000
** **Strong	196	35	1.00	1.00	
Patient satisfaction					
** **Satisfied	219	95	1.00	1.00	
** **Unsatisfied	118	86	1.68(1.16-2.43)	2.03(1.24-3.31) **	0.002

* - P-value of 0.01–0.05; ** - P-value of 0.01–0.001; *** - P-value of less than 0.001; COR – Crude Odds Ratio; AOR – Adjusted Odds Ratio; CI – Confidence Interval; ETB – Ethiopian Birr.

Participants with the age groups of 36–50 and 50–65 years were more than two and fourfold more likely to have depressive symptoms than those under the age of 36 (AOR = 2.60, 95% CI: 1.13–6.02) and (AOR = 4.09, 95% CI: 1.79–9.36), respectively. Compared to being currently married, being currently unmarried raised the odds of developing depressive symptoms by six times (AOR = 5.95, 95% CI: 3.37–10.50). Moreover, the social support acquired by the study subject, compared to study subjects getting strong social support, those getting low and moderate support were more than six (AOR = 6.73, 95% CI: 3.65–12.42) and four times (AOR = 4.04, 95% CI: 2.22–7.36) higher odds of experiencing depressive symptoms, respectively.

Regarding the level of VI, the odds of experiencing some level of depressive symptoms by severely visually impaired study participants was almost three times (AOR = 2.98, 95% CI: 1.43–6.23) higher than those living with mild VI. Compared to their counterparts, the odds of being depressed was more than threefold higher in the study subjects that had undergone an ocular surgery (AOR = 3.60, 95% CI: 1.93–6.74) and that had a sudden vision loss pattern (AOR = 3.51, 95% CI: 2.04–6.03). Furthermore, dissatisfaction with the service delivered by the tertiary eye care center doubled the likelihood of depressive symptoms occurrence (AOR = 2.03, 95% CI: 1.24–3.31).

### Factors associated with anxiety symptoms

Sex, religion, occupation, duration of VI, current status of VI, history of systemic illness, and history of substance use had a p-value of > 0.2, and they did not get fitted to multivariable logistic regression for anxiety symptoms. In the multivariable logistic regression model, living circumstances, pattern of vision loss, history of eye surgery, level of VI, social support, and patient satisfaction were found to have a significant association with anxiety symptoms at a p-value < 0.05 (Table 5).

**Table 5:** Factors associated with anxiety symptoms among adult visually impaired follow-up patients attending HUCSH-ECTC, South Ethiopia, 2024 (n = 518).

Variables	Anxiety symptoms		COR (95%)	AOR (95%)	P-value
	No	Yes			
Age					
** **18-35	72	52	1.00	1.00	
** **36-50	88	32	0.50(0.29-0.86)	0.86(0.37-2.00)	0.090
** **51-65	84	57	0.94(0.58-1.53)	1.03(0.45-2.37)	0.183
** ** > 65	70	63	1.25(0.76-2.04)	0.96(0.37-2.48)	0.069
Residence					
** **Urban	213	108	1.00	1.00	
** **Rural	101	96	1.88(1.30-2.70)	0.64(0.31-1.33)	0.065
Marital status					
** **Currently married	207	100	1.00	1.00	
** **Currently not married	107	104	2.01(1.40-2.89)	1.20(0.65-2.18)	0.080
Educational status					
** **Non-formal education	90	81	1.64(1.13-2.38)	0.99(0.50-1.99)	0.061
** **Formal education	224	123	1.00	1.00	
Living circumstance					
** **First degree relative	265	149	1.00	1.00	
** **Alone	33	18	0.97(0.53-1.78)	0.75(0.32-1.78)	0.159
** **Relatives	16	37	4.11(2.21-7.64)	2.61(1.13-6.03) *	0.032
Valid health insurance					
** **Yes	180	94	1.00	1.00	
** **No	134	110	1.57(1.10-2.24)	1.38(0.81-2.36)	0.095
Average family monthly income (in ETB)					
** ** < 4000	44	55	3.11(1.76-5.47)	1.51(0.62-3.69)	0.365
** **4000-6000	131	91	1.73(1.06-2.80)	1.13(0.58-2.23)	0.239
** **6000-8000	57	25	1.09(0.59-2.03)	1.33(0.60-2.96)	0.118
** ** > 8000	82	33	1.00	1.00	
Family size					
** **3-4	110	53	1.00	1.00	
** **5-6	166	127	1.59(1.06-2.37)	1.61(0.95-2.72)	0.220
** ** > 6	38	24	1.31(0.71-2.41)	1.13(0.51-2.50)	0.132
Pattern of vision loss					
** **Sudden	70	118	4.78(3.26-7.03)	6.83(4.12-11.32) ***	0.000
** **Progressive	244	86	1.00	1.00	
Additional impairment physical and sensory					
** **Yes	5	12	3.86(1.34-11.13)	0.82(0.19-3.48)	0.231
** **No	309	192	1.00	1.00	
History of eye surgery					
** **Yes	46	54	2.10(1.35-3.26)	3.94(2.19-7.12) ***	0.000
** **No	268	150	1.00	1.00	
Level of VI					
** **Mild	113	40	1.00	1.00	
** **Moderate	161	86	1.51(0.97-2.36)	1.89(1.09-3.30) *	0.040
** **Severe	40	78	5.51(3.26-9.31)	4.59(2.35-8.96) ***	0.000
Social support					
** **Poor	61	92	5.90(3.75-9.31)	4.13(2.16-7.86) ***	0.000
** **Moderate	69	65	3.69(2.31-5.88)	3.69(2.09-6.51) ***	0.000
** **Strong	184	47	1.00	1.00	
Patient satisfaction					
** **Satisfied	220	94	1.00	1.00	
** **Unsatisfied	94	110	2.74(1.90-3.95)	2.77(1.73-4.42) ***	0.000

* - P-value of 0.01–0.05; ** - P-value of 0.01–0.001; *** - P-value of less than 0.001; COR – Crude Odds Ratio; AOR – Adjusted Odds Ratio; CI – Confidence Interval; ETB – Ethiopian Birr.

This empirical study confirmed that living with relatives doubled the occurrence of anxiety symptoms compared to being with first-relative families (AOR = 2.61, 95% CI: 1.13–6.03). Likewise, acquiring moderate (AOR = 3.69, 95% CI: 2.09–6.51) or less (AOR = 4.13, 95% CI: 2.16–7.86) social support raised the odds of being anxious by more than threefold compared to being strongly supported.

The odds of experiencing some level of anxiety symptoms among individuals with moderate and severe VI were nearly 2 (AOR = 1.89, 95% CI: 1.09–3.30) and above 4 times (AOR = 4.59, 95% CI: 2.35–8.96) higher than those with mild VI, respectively. Additionally, compared to its comparator, having a history of ocular surgery upraised being anxious by about fourfold (AOR = 3.94, 95% CI: 2.19–7.12). Adult visually impaired patients who had a sudden vision loss had more than 6 times (AOR = 6.83, 95% CI: 4.12–11.32) higher anxiety symptoms than individuals who lost their vision progressively. Alike to what was observed on depressive symptoms, study subjects unsatisfied with the eye care service being obtained were two times more likely to experience some level of anxiety symptoms (AOR = 2.77, 95% CI: 1.73–4.42).

## Discussion

Especially for developing nations, the burden of common mental disorders, such as depressive and anxiety symptoms, is getting the public health concern and becomes a double burden over the established infectious diseases. Understanding the actual burden on different segments of the community could be used as baseline data to catch the eyes of policymakers, leading to modified, efficient, and evidence-based clinical practice resulting in improved quality of life. This facility-based study covering a large sample size assessed the burden of depressive and anxiety symptoms, and their associated factors among adult, visually impaired follow-up patients.

In this study, the magnitude of depressive symptoms in adult follow-up patients suffering from VI was found to be 34.9% (95% CI: 29.3–41.3). This finding was in line with the previous studies conducted in Ethiopia (30.6%) [23], Nigeria (34.1%) [41], New Zealand (29.4%) [42], and the Netherlands (32.2%) [28]. The socioeconomic status similarity would be mentioned as a possible reason for alignment with a study from Africa [43]. Depressive symptoms burden showed significant and consistent correlation with socioeconomic status of the nation, family and individuals as well. Therefore having the same socioeconomic status could result comparable burden of depressive symptoms. Moreover, a report from Netherlands also enrolled comparable population with the same level of visual impairment with this study, which resulting quite similar burden [28].

However, the result of this study was higher than the reports from Ethiopia (26.7%) [22], Nigeria (8.7–27.1%) [19,44,45], Asia (12% and 28%) [46–49], the United States (10.7%) [50], Australia (14.7%) [51], and Europe (9.4%−22.8%) [24,25,52]. This discrepancy could be due to the depressive symptoms measurement tool, data collection technique, study population, and socio-economical difference [53]. For instance, an Ethiopian, Nigerian, and variety of the study were conducted on older adults having VI; this condition potentially underestimated the burden of depressive symptoms on the previous study. Nowadays, depressive symptoms is getting more prevalent in adults compared with those having more than 60 years of age [25,54]. This phenomenon incriminates that sufficient care helps adult visually impaired population to cope with this desperating condition. In the Norway study, the data were gathered via telephone interviews causing reluctance to respond each details of the questions as the face-to-face one collected in the health care center. Additionally, studies carried out in Europe, Asia, USA, and Australia were conducted in middle- and high-income nations with established and integrated eye and health care system resulting with more controlled burden of common mental disorders like depressive symptoms [55]. Additionally, significant fall down of mortality rate was also observed among those getting adequate health care. This finding suggests that early detection and treatment of depressive symptoms in visually impaired people leads to better depressive symptoms control and a higher quality of life.

Conversely, the finding of this study was lower than studies conducted in Pakistan (56%) [27] and Nigeria (50%) [20]. The inconsistency could be due to the variations in clinical characteristics of the study population and measurement tools used for depressive symptoms measurement. Participants in the Nigerian study had moderate to severe VI, which increased their functional, social, and financial burden resulting, boosted burden of depressive symptoms [27]. Furthermore, the HADS scale was applied in this investigation to measure depressive symptoms, with a cutoff point of 8 out of 21 total scores for depressive symptoms; in contrast, the Pakistan study employed the Centre for Epidemiologic Studies-Depressive symptoms scale (CES-D) with a cutoff point of 10 out of 60 total scores. Therefore, in comparison to this study, the previous results were expected to show a higher prevalence of depressive symptoms.

To be consistent with the study from Nigeria (37.5%) [41], the magnitude of anxiety was found to be 39.4% (95% CI: 35.1–43.8) in this study. The relatively similar socio-economic status and the similarity of the tool used for anxiety symptoms measurement would be attributable for this occurrence.

This burden of anxiety symptoms was relatively higher than observed in Nigeria (29.5%) [29,30], (10.4%) [21,29], Malaysia (8%−17%) [48], the Netherlands (15.6%) [28], the United States (27.2%) [56], France – Germany – Italy (30.1%) [57], and China (13.5%) [47]. This discrepancy may result from the fact that different studies use different tools having variety of reliability, consistency, classification, and detailed assessment causing inconsistency in estimation of the burden of depressive symptoms. Moreover, the way and area of data collection affecting the motivation and the social desirability challenge could influence the outcome. However, as the reported anxiety symptoms levels were from various socioeconomic nations across the globe, it is reasonable that there would be a variance in the prevalence of anxiety symptoms due to socioeconomic disparities [58]. The common mental disorders among patients in the health care centers could better addressed early and managed appropriately in developed nations such as Europe and Far East Asia [59].

The present study yielded a lower result than the previous study conducted in Nigeria, (53.2%) [20]. This discrepancy was may be attributed to differences in the study population, whereby the former study included patients with a more severe level of VI, while this study included patients with a mild to severe level of VI. Consequently, higher prevalence was observed in Nigerian study than us [22]. Therefore giving more attention for more advanced VI is expected to deal with their problems and make them hopeful and productive.

The odds of having depressive symptoms among participants those of older age groups (36–50 years and 51–65 years) was significantly higher than young age adults, making it diverged from an outputs from Norway and Britain [24,25]. A clinical research has demonstrated that growing older is frequently linked to a number of age-related eye disorders. Additionally, growing older on its own increases depressive symptoms because it limits daily activities and introduces psychosocial stressors like retirement, loneliness, and the death of a loved one [60,61]. Likewise, as compared to their comparators the currently unmarried participants had significantly higher odds of developing depressive symptoms. This condition was in agreement with the reported from Ethiopia, Nigeria, Armenia, the Netherlands, and Britain [26]. Being currently single is one of the most devastating life events that are most associated with negative stress among visually impaired patients in particular report less well-being and more symptoms of depressive symptoms compared to married individuals [62]. Especially by the time of challenges, marriage also creates an opportunity to share ideas and support each other helping to cope with the condition [63].

The odds of having depressive symptoms in participants who had poor and moderate social support were 6.73 and 4.04 times higher than in participants who had strong social support respectively. This result was comparable with the studies conducted in Ethiopia, Nigeria, Australia, Pakistan, and Armenia [26]. Prior studies have found that visually impaired patients concerning social support, it is more plausible that low level of received social support will lead to an increased likelihood of loneliness. Again, the vision loss is also causing isolation and loneliness. Furthermore, their families and community do not understand the psychosocial impact of the vision loss, and not support them. These reasons again cause the frequent occurrence of depressive symptoms [60]. Taking care of visually impaired individuals who are working and living around us is vital in protecting them from experiencing some levels of common mental disorders. Similarly, to be in accordance with reports from Ethiopia, Nigeria, and Pakistan [26], severely visually impaired subjects were almost threefold more likely to be depressed than those living with low level VI. This phenomenon was occurred because of the fact that he severe VI hinders the social, functional, and economic disability resulting an individual lonely, desperate, and depressed [22,64].

In contrary with an evidence from Norway [25], depressive symptoms was highly observed in visually impaired patients who lost their vision suddenly. Sudden loss of vision can lead to unfortunate socio-economic disturbance like losing financial leadership and source of income. Usually, this occurs in an unexpected manner that might put the person under stress and leading to depressive symptoms [65]. This result underlines the advantage of the detailed examination of the visual status with the corresponding coping mechanisms employed to deal with the VI they suffering from. Routine monitoring of the psychosocial health with the primary ocular and visual checkup is recommended. The odds of having depressive symptoms among participants who had a history of eye surgery was 3.60 times higher than participants who had no history of surgery. This link explained by surgical therapy associated with result expectancies and fear of complications [66]. Spending adequate time to explain the potential prognosis of the procedure could help the patient to arrange his/her expectation accordingly.

Regarding the eye care delivery, dissatisfaction with an eye care services was observed to double the occurrence of depressive symptoms among adult, visually impaired follow-up ophthalmic patients. Patients who are more satisfied with the eye care services they receive are more likely to follow their treatment plan, take better care of their health, and lead healthy lives. This determines better outcome of their treatment and reduced occurrence of depressive symptoms [67]. Working hard in improving the quality of the service being given and reducing the waiting time will make follow-up patients more satisfied with the service and have healthy psychosocial health.

The odds of having anxiety symptoms in participants who had moderate and severe levels of VI were 1.89 and 4.59 times higher than in participants who had a mild level of VI, respectively. This result is comparable with the studies conducted in Nigeria and Japan [29,30]. Daily activities may become more difficult when visual acuity declines, which could have negative effects on social and economic standing, increase dependency, raise the risk of falling, and result in poor mental well-being and anxiety [68]. Additionally, this study confirmed that getting moderate or less social support raised experiencing anxiety symptoms significantly compared to acquiring strong social support. This result was in convergence with the studies conducted in Nigeria [29]. Prior studies have found bidirectional relations between social support and anxiety symptoms; increased perceptions of social support led to increased self-efficacy and decreased avoidance, which in turn leads to decreased anxiety symptoms. Increases in support perception may arise from changes in resources or cognitions, developing more social skills that may lead to improvements in anxiety symptoms [69].

The likelihood of anxiety symptoms among participants with a sudden pattern of vision loss was 6.83 times higher than among participants with a progressive pattern of vision loss. Sudden loss of vision can lead to unfortunate socio-economic disturbance like losing financial leadership and source of income. Usually, this occurs in an unexpected manner that might put the person under stress [65]. Individuals who lived with relatives had 2.61 times the likelihood of having anxiety symptoms as compared to those who lived with first-relative families. which agrees with research conducted in Ethiopia [23,28], and the Netherlands [23,28]. Their financial load and stress may be the cause, as well as their feelings of rejection and low status. A previous study reported that the psychological well-being of individuals who lived alone or with others was poorer than those who lived with first-relative families; those can provide a sense of well-being and emotional support, producing mutual obligations and reinforcements between them [70].

Study participants with a history of eye surgery were 3.94 times more likely to have anxiety symptoms as compared with those who did not have a history of surgery. This might be due to emotional and physiological reactions to the anticipated surgery, which might have indirectly influenced the results. In addition to this, the high cost of treatment, poor quality of care, an increase in the length of hospital stays, and increases in hospital readmission rates due to surgical treatment [71]. The odds of having anxiety symptoms among participants who were unsatisfied with the eye care service they provided were 2.77 times higher than participants who were satisfied with the eye care service they provided. The increased satisfaction with the provided eye care health services is important as the patients are satisfied with the consultation and more likely to adhere to their treatment plan, take better care of their health, and lead a general well-being of the patient, especially mental health aspects [67].

## Strength and limitation of the study

This study encountered some limitations such as recall and social desirability bias. The outcome may be subject to social desirability and recall bias due to the self-reported interview used to address several important socio-demographic, economic, and other characteristics of the study subjects. Appropriate correction was done on optimizing the recall time for patients and strict and detail instruction and clarification for the patient before data collection, to address some challenges identified during the pre-test. A thorough explanation was provided, covering topics such as data security, anonymity, safe output communication, and interview room privacy. Moreover, the cross-sectional nature of the study potentially hinders the assessment of the time-based status of depressive and anxiety symptoms. It does not also show the cause-and-effect relationship between dependent and independent variables assessed. This study also fails to assess some potentially important factors including detailed lifestyle and behavioral status, functional disability, wealth status, and systemic comorbidity among the study subjects, which could affect the conclusion and generalizability of the results. Furthermore, limited generalizability of the results of this study is observed. Since this study was conducted at a specified setting, generalizing results to the wider general population is not possible. Anticipating future researches addressing those limitations of the study will complement the quality of this output and help the clinical practice. Nonetheless, using validated tool to assess depressive and anxiety symptoms is one of strength of the study, the symptom-based tools we used only screen the depressive and anxiety symptom based mental conditions; not actually meaning depressive and/or anxiety disorder is observed. We advise researchers consider more clinical and objective techniques based investigation of common mental disorders among this vulnerable population, especially in a resource-constrained areas. Additionally, those study subjects confirmed to live with depressive or anxiety symptoms were linked to the psychiatry clinic of the Hawassa University Comprehensive Specialized Hospital for further investigation and timely management.

## Conclusion

In contrast to earlier researches, a significant proportion of visually impaired people attending the HUCSH-ECTC had depressive and anxiety symptoms. This finding indicated that it is crucial to integrate treatment services for co-occurring mental disorders with VI and implement evidence-based interventions. Getting poor and moderate social support, suffering from moderate and severe levels of VI, having a history of eye surgery, having a sudden pattern of vision loss, and being unsatisfied with the eye care service received were variables significantly associated with both depressive and anxiety symptoms.

## Supporting information

S1 Fig Schematic presentation of sampling technique and procedures used for assessing depressive and anxiety symptoms and their associated factors. (TIF)

S2 Fig Severity of depressive and anxiety symptoms among the study subjects. (TIF)

S1 Table Data. (XLS)

## Abbreviations

HADS: Hospital Anxiety and Depressive symptoms Scale
HUCSH-ECTC: Hawassa University Comprehensive Specialized Hospital-Eye Care and Training Center
OSSS: Oslo Social Support Scale
PSQ: Patient Satisfaction Questionnaire
PVA: Presenting Visual Acuity
VI: Visual Impairment
