# Supplementary figures and images for: Depressive and anxiety symptoms, and their associated factors among adult, visually impaired follow-up patients attending the Hawassa University Comprehensive Specialized Hospital Eye Care and Training Center, South Ethiopia, 2024
Source: PLoS One. 2025 Jul 2;20(7):e0326117. doi: 10.1371/journal.pone.0326117 (PMC12221031; doi:10.1371/journal.pone.0326117)

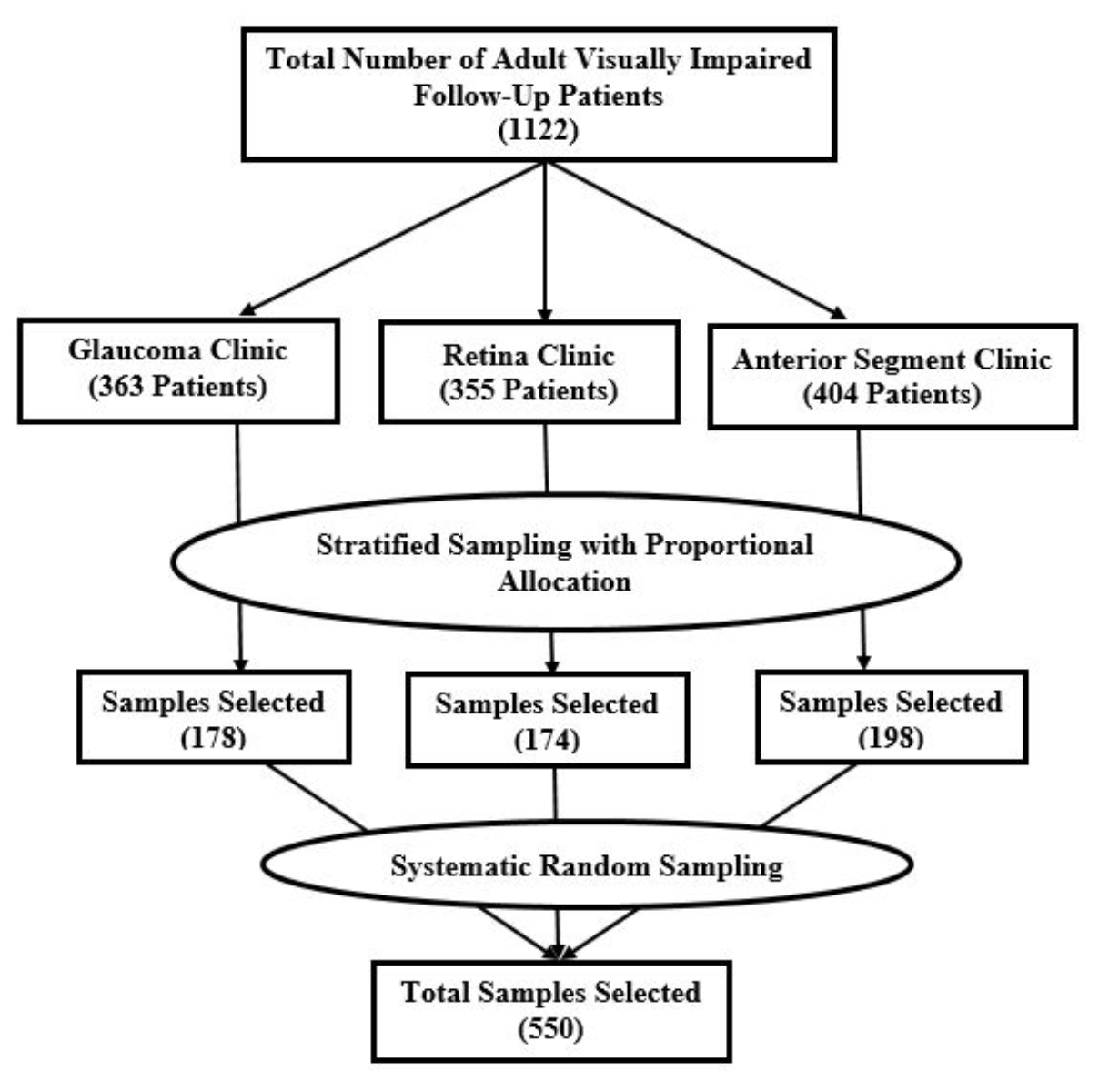

Supplement: S1 Fig — (TIF) [file pone.0326117.s001.tif]

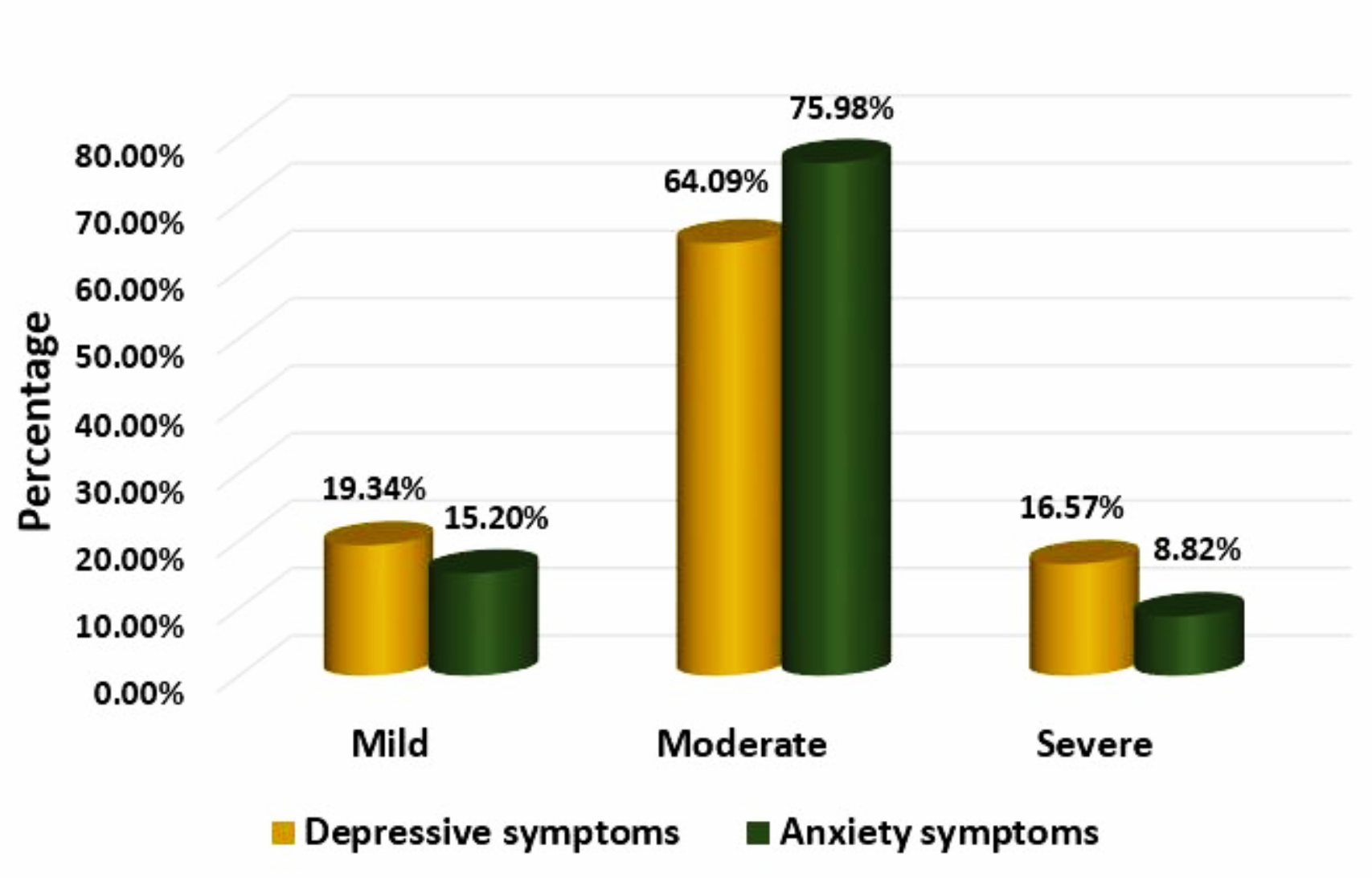

Supplement: S2 Fig — (TIF) [file pone.0326117.s002.tif]
